# Tuning Magnetic Entropy Change and Relative Cooling Power in La_0.7_Ca_0.23_Sr_0.07_MnO_3_ Electrospun Nanofibers

**DOI:** 10.3390/nano10030435

**Published:** 2020-02-29

**Authors:** Luis Andrés Burrola Gándara, Lizeth Vázquez Zubiate, Diana M. Carrillo Flores, José T. Elizalde Galindo, Carlos Ornelas, Manuel Ramos

**Affiliations:** 1 Departamento de Física y Matemáticas, Instituto de Ingeniería y Tecnología, Universidad Autónoma de Ciudad Juárez, 450N Avenida del Charro, Ciudad Juárez 32310, Mexico; lizethvazquezzubiate@gmail.com (L.V.Z.); diana.carrillo@uacj.mx (D.M.C.F.); jose.elizalde@uacj.mx (J.T.E.G.); manuel.ramos@uacj.mx (M.R.); 2 Laboratorio Nacional de Nanotecnología, Centro de Investigación en Materiales Avanzados S.C., Miguel de Cervantes 120, Complejo Industrial Chihuahua, Chihuahua C.P. 31136, Mexico; carlos.ornelas@cimav.edu.mx

**Keywords:** magnetocaloric effect, manganite, nanofibers, electrospinning

## Abstract

We present experimental evidence about the magnetocaloric tuning effect in one-dimensional nanostructure fibers mixed-valence manganite as synthesized by electrospinning techniques and under heat treatments of 973, 1073 and 1173 K. The stoichiometry obtained is La_0.7_Ca_0.23_Sr_0.07_MnO_3_ and Rietveld refinement indicates a single-phase with an orthorhombic (Pnma) crystal structure. Scanning and transmission electron microscopy observations indicate coalescence in granular colonies of La_0.7_Ca_0.23_Sr_0.07_MnO_3_ nanoparticles to conform nanofibers. Magnetic entropy change is tuned due to heat treatments at 1173 K with maximum values of 1, 1.82 and 2.51 J/kgK for applied external magnetic fields of *μ*_0_*H* = 1, 2 and 3T, respectively, with a maximum magnetic entropy difference at a Curie temperature of 293 K (furthermore, second-order magnetic phase transition was observed). Additionally, for a magnetic field, ~*μ*_0_*H* = 3 T values of 49, 95 and 143 J/kg for 973, 1073 and 1173 K heat-treated samples were obtained.

## 1. Introduction

The development of a new cooling technology referred as magnetic refrigeration, is based on the magnetocaloric effect (MCE), considered an intrinsic property of magnetic materials. MCE technology aims to replace traditional refrigeration systems mainly due to its high energy efficiency, compact design and ecological interplay with the environment [1]. When estimating MCE two parameters are considered: the isothermal entropy change and the adiabatic temperature gradient. Both take place when an external magnetic field is applied and removed from a magnetic material. The variation of the external applied magnetic field and its interaction with the magnetic material, change the magnetic contribution of total entropy and originate the magnetocaloric effect as described by the authors of [2]. The maximum value of magnetic entropy change occurs near magnetic phase transition temperature, and strongly depends of the magnetic field magnitude [3]. The relative cooling power (RCP) is a parameter used to identify a suitable magnetocaloric material for magnetic refrigeration applications. In an ideal refrigeration cycle, RCP is the amount of heattransferred between hot and cold reservoirs [4]. One main point of interest for the synthesis of nanodimensional (1D, 2D) ceramic materials, like nanofibers, nanowires and nanorods is to achieve remarkable physical properties for applications in photonics, electronics, mechanics and recently, nanostructured materials for advanced magnetism applications [5,6]. The electrospinning technique is picked to fabricate 1D nanostructure specially for ceramic nanofibers, due to its fast and low-cost route to achieve a one-dimensional nanostructure with a specific shape and chemical parameters [7]. The perovskite manganite is a relevant ceramic due to the manipulation of its critical size and the formation of multidomain, as found in the literature for La_0.6_Sr_0.4_CoO_3_ nanotubes (d~100 nm) and nanowires (d~40–60 nm) and as extensively described by Li et al. [8]. Generally, manganite complex oxides with stoichiometry R_1-x_A_x_MnO_3_ (R a trivalent rare earth element and A represents alkaline earth cations) possess magnetic properties susceptible to crystalline structure [9]. Also, double exchange interaction between Mn^3+^ and Mn^4+^ ions and strong spin-lattice coupling cause a significant magnetic entropy change near the magnetic phase transition [10]. Furthermore, R_1-x_A_x_MnO_3_ manganites are ideal materials candidates for the development of magnetic refrigeration technology, due to their substantial MCE values, preparation forms, low cost, chemical stability, and tuning of magnetic transition temperatures [11,12]. Here, we present magnetocaloric effect in La_0.7_Ca_0.23_Sr_0.07_MnO_3_ nanofibers fabricated by the electrospinning technique along with extensive characterization using X-ray diffraction, scanning and transmission electron microscopy and vibrating sample magnetometry.

## 2. Materials and Methods

The nanofibers with stoichiometry La_0.7_Ca_0.23_Sr_0.07_MnO_3_ were synthesized using La, Ca, Sr and Mn acetates as precursors. All precursors were dissolved in deionized water at 0.1 molar and subsequently mixed with 20% of polyvinylpyrrolidone (PVP) to form a homogenous solution. Using a stainless steel 22-gauge needle with working distance of 15 cm between needle and aluminum substrate, 0.1 mL/h fluid flow and 15 kV as set conditions; nanofibers were produced with deposits via a Tong Li Tech Co.^®^ electrospinning equipment model TL-Pro©. The as-deposited nanofibers were collected and subjected to heat treatments of 973 K, 1073 K and 1173 K (ramp rate of 1 K/min) for 1.5 h, as described previously by Burrola et al. [13]. Phase formation and crystal structure of the as-synthesized nanofibers were studied by X-ray diffraction (XRD) technique with a panalytical diffractometer model X’Pert Pro-MPD© located at IIT-UACJ facilities. The crystallite size (d) of the samples was calculated from Scherrer equation (d = 0.9 λ⁄(β cosθ)), where λ is the wavelength of Cu Kα (1.54059 Å), θ is Bragg angle and β is the full width at half maximum. The morphology of fibers was observed using a scanning electron microscopy (SEM) model JEOL 6010 LV^®^ and transmission electron microscopy (TEM) with a field emission 2200-JEOL^®^ equipped with CCDcamera, STEM unit, high-angle annular dark-field (HAADF) detector and X-Twin lenses. The sample was dispersed on isopropanol and drop cast onto 200 mesh Lacey Formvar Carbon TEM grids. Measurements of magnetization isotherms were carried out in a Quantum Design Versalab^®^, with a vibrating sample magnetometer attachment.

## 3. Results and Discussion

### 3.1. Powder X-ray and Rietveld Refinement

**Figure 1:**
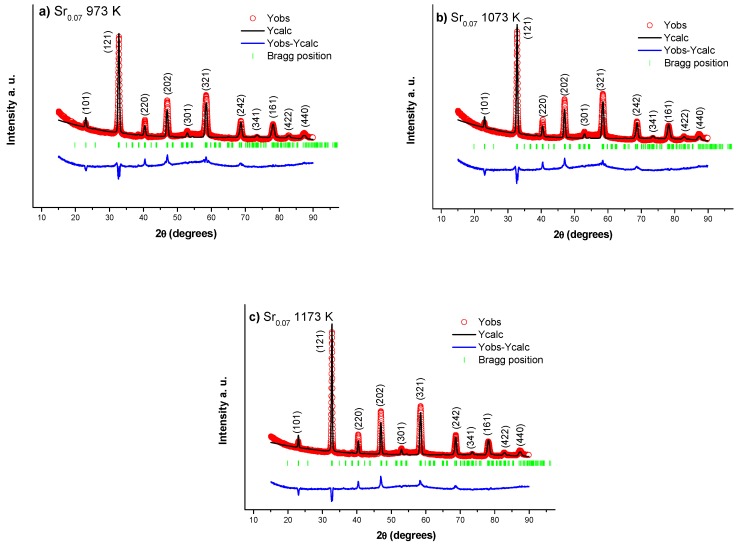
Rietveld refinements for La_0.7_Ca_0.23_Sr_0.07_MnO_3_ nanofibers heat treated at (**a**) 973 K, (**b**) 1073 K and (**c**) 1173 K correspond to single-phase orthorhombic structure (Pnma) with crystallite size of (**a**) 48 nm, (**b**) 50 nm and (**c**) 62 nm, respectively.

Using information from powder X-ray diffraction patterns performed at room temperature, the Rietveld refinements was calculated using Full Proof program. Figure 1 presents the diffraction data for La_0.7_Ca_0.23_Sr_0.07_MnO_3_ nanofibers under thermal treatment of (a) 973 K, (b) 1073 K and (c) 1173 K. The crystallographic information for all refinements was acquired using 56636 ICSD card, which corresponds to single-phase orthorhombic structure Pnma space group and crystallite size of 48 nm 50 nm and 62 nm for (a) 973 K, (b) 1073 K and (c) 1173 K, respectively. One can conclude that crystallite size increases due to heat treatments, possibly due to coalescence phenomena, as confirmed by TEM measurements. A summary of Rietveld refinement is presented in Table 1, corresponding to lattice parameters, cell volume and crystal structure for samples under thermal treatment of 973 K, 1073 K and 1173 K.

### 3.2. High-Resolution Transmission and Scanning Electron Microscopy

**Figure 2:**
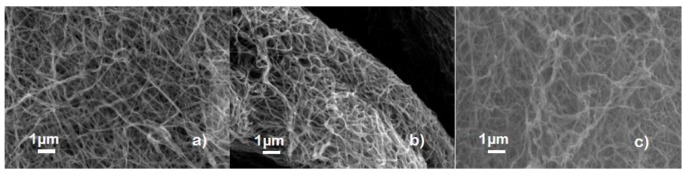

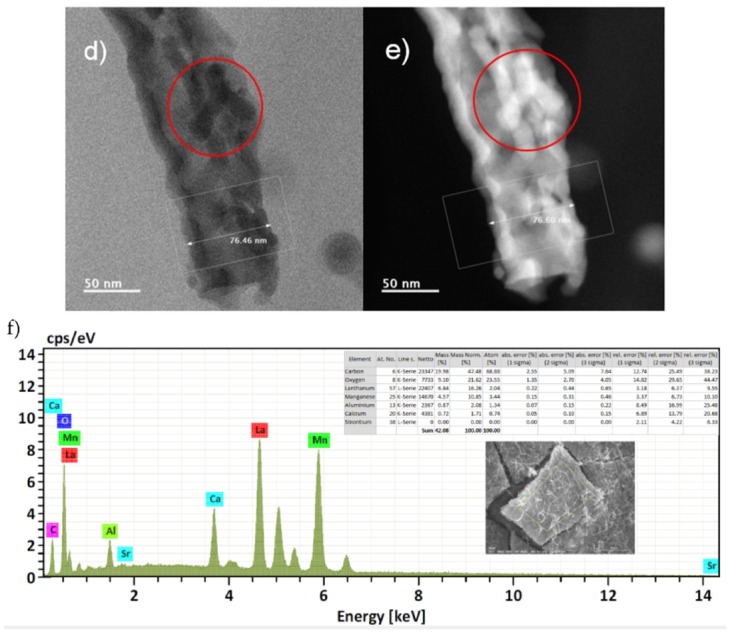
Top: scanning electron micrographs for La_0.7_Ca_0.23_Sr_0.07_MnO_3_ nanofibers at (**a**) 973 K, (**b**) 1073 K and (**c**) 1173 K. Bottom: (**d**,**e**) Scanning transmission electron micrographs for La_0.7_Ca_0.23_Sr_0.07_MnO_3_ nanofiber in dark and bright field modes (Z-contrast); red circle denotes coalescence effect among granular colonies of La_0.7_Ca_0.23_Sr_0.07_MnO_3_. (**f**) Energy-dispersed X-ray spectra and chemical count analysis.

In order to determine morphology and size of La_0.7_Ca_0.23_Sr_0.07_MnO_3_ nanofibers, transmission and scanning electron microscopy were applied. Results indicate nanofibers and agglomeration with no beads fomation, which is typical due to injection rate (0.1 mL/h) of the as-synthesized solution, along with thermal treatments, as observed clearly in Figure 2. From scanning transmission electron (Z-contrast), it was possible to observe coalescence effect of La_0.7_Ca_0.23_Sr_0.07_MnO_3_ nanocrystallites to conform nanofibers, in agreement with Zhou et al. [5] and as presented in Figure 2.

### 3.3. Magnetic Entropy Change

**Figure 3:**
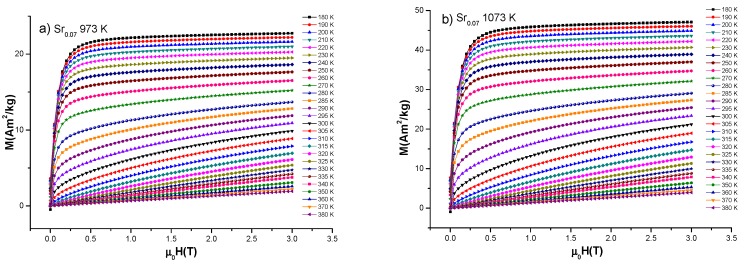

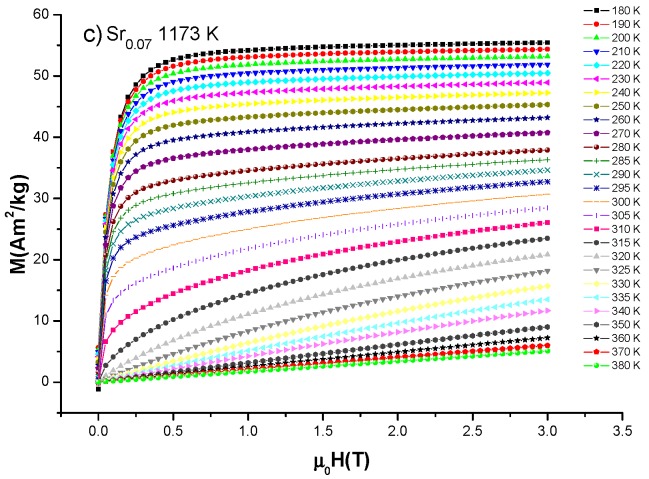
Magnetization isotherms for La_0.7_Ca_0.23_Sr_0.07_MnO_3_ nanofibers heat treated at (**a**) 973 K, (**b**) 1073 K and (**c**) 1173 K under applied field of *μ_o_H* = 3T.

From curves of magnetization as a function of magnetic field measured at constant temperatures, magnetic entropy change was estimated for the three samples. The measurements were carried out in temperature range 180–380 K and under applied magnetic field up to *μ_o_H* = 3T. Results of magnetization isotherms for (a) 973 K, (b) 1073 K and (c) 1173 K are presented in Figure 3, in which magnetization dependence of temperature is observed. The magnetization in M-H curves shows a ferromagnetic behavior for low isothermal temperatures and a paramagnetic behavior for high isothermal temperatures, which is related to a ferromagnetic to paramagnetic (FM-PM) transition around Curie temperature (*T_C_*) [3] with corresponding increment of magnetization saturation for samples under high thermal treatments.

**Figure 4:**
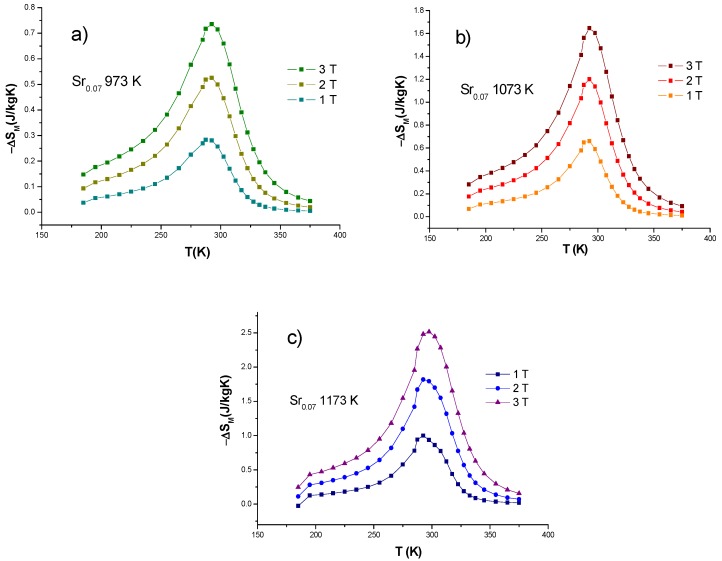
(**a**) Magnetic entropy change for thermal treatment of 973 K. (**b**) Magnetic entropy change for thermal treatment of 1073 K and (**c**) magnetic entropy change for thermal treatment of 1173 K. As measured under applied magnetic field of *μ*_0_*H* = 1, 2 and 3 T.

Using the magnetization isotherms data as obtained for all samples, the magnetic entropy change can be calculated using thermodynamic Maxwell relation, as described by Tola et al. [14], as follows:

(1) $$\left|\Delta S_{m}\left(T,H\right)\right|=\int_{0}^{H}{\left(\frac{\delta M\left(T,H\right)}{\delta T}\right)}_{H}dH$$

where *δM* is change on magnetization, and *T* and *H* are temperature and applied magnetic field, respectively. Due the discrete data of isothermal magnetization acquired from experimental measurements, a numerical approach to thermodynamic Maxwell relation using Equation (2) as described by Khlifa et al. [15] is taken, as follows:

(2) $$\Delta S_{M}\left(T,\;\mu_{0}H\right)=\sum \left(\frac{M_{i}-M_{i+1}}{T_{i+1}-T_{i}}\right)\Delta \mu_{0}H_{i}$$

where *M_i_* and *M_i+_*_1_ are the magnetization values measured at *T_i_* and *T_i+_*_1_ temperature, respectively, for a corresponding applied magnetic field *μ*_0_*H_i_*. Moreover, to identify any efficiency of a magnetocaloric material in a refrigeration cycle, it is important to consider the refrigerant cooling power (RCP) parameter. The expression to obtain RCP is displayed by Equation (3), in agreement with Iqbal et al. [16]:

(3) $$RCP=\Delta S_{M}^{max}\times \delta T_{FWHM}$$

where ∆*S_M_^max^* is the maximum magnetic entropy change value and *δT_FWHM_* the full width at half maximum of the magnetic entropy curve. The magnetic entropy change as a function of temperature is presented in Figure 4 for sample under thermal treatment of 973 K. The curves are depicted for magnetic fields applied of *μ*_0_*H* = 1, 2 and 3 T, where a maximum magnetic entropy change occurs near 293 K and remains invariable when applied magnetic field increases. This temperature is pointed out as *T_C_*, where the magnetic phase changes from a ferromagnetic to a paramagnetic state as discussed for Figure 3. Besides, an increase of the magnetic entropy change is noticed as the magnetic field increases from *μ*_0_*H* = 0 T to 3 T.

This behavior can be described with the Maxwell relation expressed in Equation (1), as the change in magnetization with respect to temperature is proportional to the magnetic field applied. The maximum values of magnetic entropy change for sample at 973 K are 0.28, 0.53 and 0.74 J/kgK for applied magnetic fields of *μ*_0_*H* = 1, 2 and 3 T respectively, as presented in Figure 4a. On the other hand, the RCP obtained increases with applied magnetic field, having values of 15, 32 and 49 J/kg in presence of external fields of *μ*_0_*H* = 1, 2 and 3 T.

Figure 4b presents data of magnetic entropy change for sample under thermal treatment of 1073 K. It was possible to determine an increment of MEC as function of applied magnetic field, having maximum values of 0.66, 1.20 and 1.65 J/kgK for applied magnetic fields of 1, 2 and 3 T. Those maximum MEC values remain for T_C_~293 K and increase when compared with, sample under thermal treatment of 973 K, which is strongly correlated with coalescence among colonies of La_0.7_Ca_0.23_Sr_0.07_MnO_3_ to conform nanofibers. Also, RCP increases with values of 29, 62 and 96 J/kg found for applied magnetic fields of 1, 2 and 3 T. At last, MEC values for sample at 1173 K are displayed in Figure 4c, with maximum magnetic entropy changes of 1, 1.82 and 2.51 J/kgK and RCP maximum values of 46, 94 and 143 J/kg, which determine that La_0.7_Ca_0.23_Sr_0.07_MnO_3_ nanofibers under thermal treatment (1173 K) possess maximum values of magnetic entropy change and RCP, when compared to the other two samples at 973 K and 1073 K, respectively. The latter are attributed to higher applied temperature and total time of heat treatment due to granule interconnexion from coalescence of La_0.7_Ca_0.23_Sr_0.07_MnO_3_, attributed to nanoscale phenomena, which improvesmagnetic homogeneity causing long-range ferromagnetic order, as extensively described for manganites by Baaziz et al. [17] and Tang et al. [18]. 

**Figure 5:**
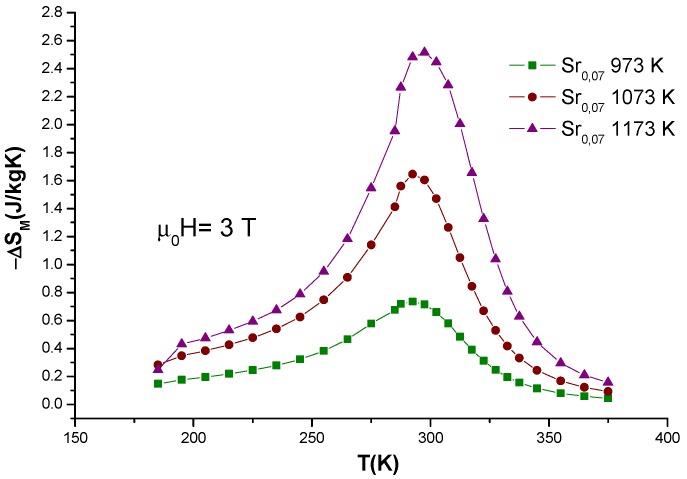
Magnetic entropy change for applied magnetic field of *μ*_0_*H* = 3 T for La_0.7_Ca_0.23_Sr_0.07_MnO_3_ nanofibers. It is possible to observe relative cooling power (RCP) increment by the broad shape on all curves.

The results for ∆*S_M_^max^*, *δT_FWHM_* and RCP parameters for all samples are summarized in Table 2 according to magnetic fields applied of *μ*_0_*H* = 1, 2 and 3 T. An increase of magnetic entropy change and RCP is clearly observed, caused by heat treatments and confirmed when applied magnetic field is present. When closely analyzing the *δT_FWHM_* parameter, it is possible to prove that −*∆S_M_* peak narrows as processing temperature increases. Interestingly, RCP increases even when the *δT_FWHM_* becomes smaller if the values are compared for same applied magnetic field, this could be attributed to an improvement of magnetic homogeneity and long-range ferromagnetic order [5]. A comparison of the magnetic entropy change in the three samples is presented in Figure 5, but only for data corresponding to applied magnetic field of *μ*_0_*H* = 3T.

In addition, Gd metal represents the benchmark material for refrigeration at room temperature because of its suitable magnetocaloric and RCP values, as extensively described by Khlifa et al. [3,15]. The maximum RCP obtained in this work is 143 J/kg, which represents a sizable value compared with 187 J/kg (*μ*_0_*H* = 2 T) or 120 J/kg (*μ*_0_*H* = 2 T), both for Gd element, as described extensively in the literature by Wang et al. [19] and by Pecharsky and Gschneidner [20], respectively.

**Figure 6:**
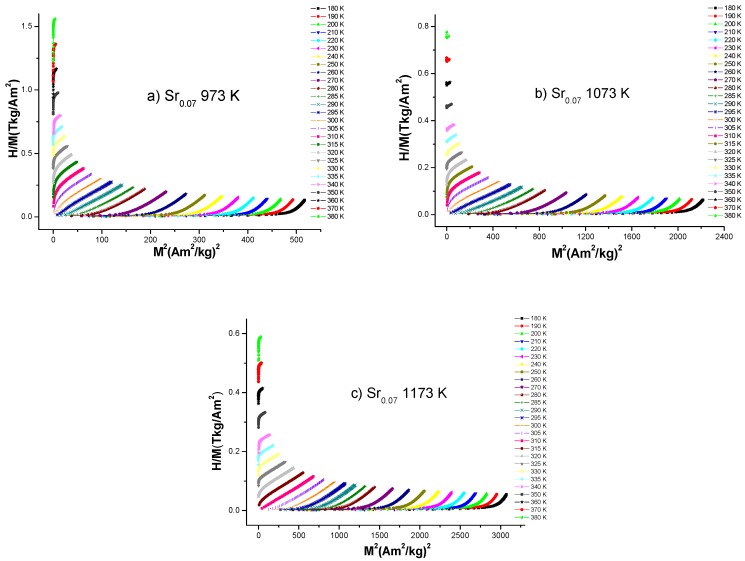
The Arrot plots for all La_0.7_Ca_0.23_Sr_0.07_MnO_3_ nanofibers subjected to thermal treatment of (**a**) 973 K, (**b**) 1073 K and (**c**) 1173 K, respectively. It was possible to observe a positive value on the slopes corresponding to a second order magnetic phase transition.

The magnetic phase transition order for all samples was determined by Arrot plots (*μ*_0_*H*⁄*M* vs. *M*^2^) displayed in Figure 6. These plots were obtained from magnetization isotherms data shown in Figure 3. Based in Banerjee criterion, a positive or negative slope in $\mu_{0}H/M$ vs. $M^{2}$ curves determine the order of the magnetic phase transition. A positive slope indicates a second-order magnetic phase transition, whereas a negative slope indicates a first order magnetic phase transition [21]. From the data obtained, a positive slope is noticed for La_0.7_Ca_0.23_Sr_0.07_MnO_3_ nanofibers with heat treatments at 973 K, 1073 K and 1173 K, which is a proof of second-order magnetic phase transition. The second-order nature of the magnetic phase transition around room temperature indicates that no thermal or magnetic hysteresis are present for this system [13], which is favorable for magnetic refrigeration. Hence, according to the results exposed, La_0.7_Ca_0.23_Sr_0.07_MnO_3_ nanofibers can be considered an interesting alternative for magnetic refrigeration at room temperature.

## 4. Conclusions

A successful synthesis of La_0.7_Ca_0.23_Sr_0.07_MnO_3_ nanofibers was obtained using the electrospinning technique. An orthorhombic single-phase crystal structure (Pnma) was identified on samples under thermal treatment of 973, 1073 and 1173 K, by Rietveld refinement. Magnetic entropy change and RCP increases as function of heat treatment temperature. Then, magnetic homogeneity was improved via coalescence effect and a long range ferromagnetic order came up sequentially with higher temperatures. Maximum magnetic entropy change values of 0.74, 1.65 and 2.51 J/kgK for 973, 1073 and 1173 K, respectively were obtained near Curie temperature of ~293 K. The relative cooling power (RCP) with maximum values of 49, 95 and 143 J/kg was obtained for 973, 1073 and 1173 K samples, respectively. A second-order magnetic phase transition was determined according to Banerjee criterion in Arrot plots. The results demonstrate that the dimensionality and composition of La_0.7_Ca_0.23_Sr_0.07_MnO_3_ nanofibers can be convenient for magnetic refrigeration at room temperature and low magnetic fields, and for potential applications in low dimension electronics, sensors and other important devices.

## Figures and Tables

**Table 1:** Summary of lattice parameters, cell volume and crystal structure obtained from Rietveld refinement for La_0.7_Ca_0.23_Sr_0.07_MnO_3_ nanofibers at 973, 1073 and 1173 K.

	a (Å)	b (Å)	c (Å)	Volume (Å)	Structure
**Sr_0.07_ (973 K)**	5.4540 (7)	7.7096 (10)	5.4992 (7)	231.23 (5)	Orthorhombic
**Sr_0.07_ (1073 K)**	5.4549 (7)	7.7087 (10)	5.4961 (7)	231.11 (5)	Orthorhombic
**Sr_0.07_ (1173 K)**	5.4582 (4)	7.7111 (6)	5.4915 (4)	231.13 (3)	Orthorhombic

**Table 2:** Summary of magnetocaloric parameters for La_0.7_Ca_0.23_Sr_0.07_MnO_3_ nanofibers under thermal treatment of 973, 1073 and 1173 K, respectively.

	*μ*_0_*H* (T)	−∆*S_M_^max^* (J/kgK)	*δT_FWHM_* (K)	RCP (J/kg)
**Sr_0.07_ (973 K)**	1	0.28	53.63	15
	2	0.53	60.45	32
	3	0.74	66.24	49
**Sr_0.07_ (1073 K)**	1	0.66	43.82	29
	2	1.2	51.87	62
	3	1.65	58	96
**Sr_0.07_ (1173 K)**	1	1	45.64	46
	2	1.82	51.74	94
	3	2.51	56.82	143
